# Supplementary material for: Rapid bacterial identification and resistance detection using a low complexity molecular diagnostic platform in Zimbabwe
Source: PLOS Glob Public Health. 2025 Apr 9;5(4):e0004343. doi: 10.1371/journal.pgph.0004343 (PMC11981161; doi:10.1371/journal.pgph.0004343)

S2 Fig. Number of organisms identified by Biofire according to department for all 780 samples tested using BCID2.


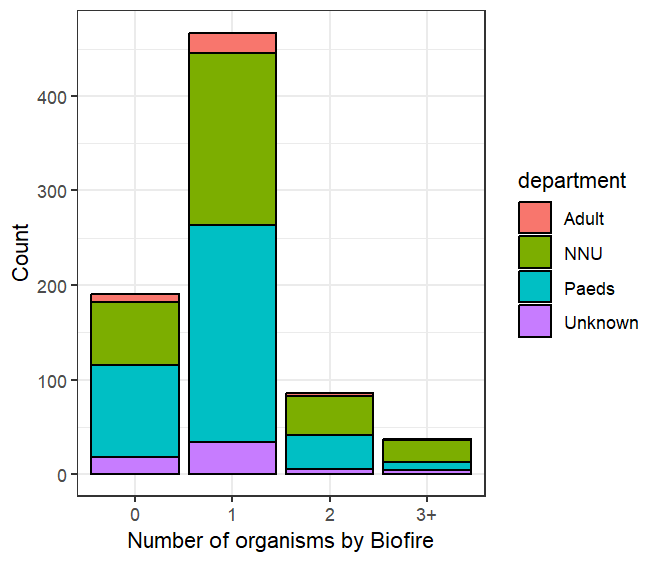

Supplement: S2 Fig — (DOCX) [file pgph.0004343.s004.docx]
