# Supplementary material for: Rapid bacterial identification and resistance detection using a low complexity molecular diagnostic platform in Zimbabwe
Source: PLOS Glob Public Health. 2025 Apr 9;5(4):e0004343. doi: 10.1371/journal.pgph.0004343 (PMC11981161; doi:10.1371/journal.pgph.0004343)
Supplement: S1 Table — (DOCX) [file pgph.0004343.s005.docx]

S1Table. Distribution of organisms detected by BCID2 according to ward for all isolates tested.

| **Biofire organism identification** | **Overall**  N = 780 | **Neonatal unit**  N = 314 | **Paediatrics**  N = 371 | **Adult**  N = 34 | **Unknown**  N = 61 |
| --- | --- | --- | --- | --- | --- |
| *Escherichia coli* | 26 (3.3%) | 12 (3.8%) | 9 (2.4%) | 5 (15%) | 0 (0%) |
| *Klebsiella pneumoniae group* | 105 (13%) | 80 (25%) | 18 (4.9%) | 0 (0%) | 7 (11%) |
| *Klebsiella oxytoca* | 18 (2.3%) | 8 (2.5%) | 10 (2.7%) | 0 (0%) | 0 (0%) |
| *Klebsiella aerogenes* | 1 (0.1%) | 1 (0.3%) | 0 (0%) | 0 (0%) | 0 (0%) |
| *Enterobacter cloacae* complex | 16 (2.1%) | 7 (2.2%) | 7 (1.9%) | 2 (5.9%) | 0 (0%) |
| *Serratia marcescens* | 4 (0.5%) | 3 (1.0%) | 1 (0.3%) | 0 (0%) | 0 (0%) |
| *Proteus spp.* | 2 (0.3%) | 1 (0.3%) | 1 (0.3%) | 0 (0%) | 0 (0%) |
| *Salmonella* spp. | 9 (1.2%) | 2 (0.6%) | 6 (1.6%) | 0 (0%) | 1 (1.6%) |
| *Acinetobacter calcoaceticus-baumannii* complex | 8 (1.0%) | 3 (1.0%) | 2 (0.5%) | 1 (2.9%) | 2 (3.3%) |
| *Pseudomonas aeruginosa* | 3 (0.4%) | 1 (0.3%) | 2 (0.5%) | 0 (0%) | 0 (0%) |
| *Stenotrophomonas maltophilia* | 4 (0.5%) | 0 (0%) | 2 (0.5%) | 1 (2.9%) | 1 (1.6%) |
| *Neisseria meningitidis* | 2 (0.3%) | 0 (0%) | 2 (0.5%) | 0 (0%) | 0 (0%) |
| *Haemophilus influenzae* | 1 (0.1%) | 0 (0%) | 1 (0.3%) | 0 (0%) | 0 (0%) |
| *Coagulase-negative staphylocci* | 411 (53%) | 149 (47%) | 212 (57%) | 18 (53%) | 32 (52%) |
| *Staphylococcus aureus* | 25 (3.2%) | 12 (3.8%) | 11 (3.0%) | 0 (0%) | 2 (3.3%) |
| *Streptococcus spp* | 26 (3.3%) | 11 (3.5%) | 14 (3.8%) | 0 (0%) | 1 (1.6%) |
| *Streptococcus agalactiae* | 23 (2.9%) | 17 (5.4%) | 4 (1.1%) | 0 (0%) | 2 (3.3%) |
| *Streptococcus pneumoniae* | 4 (0.5%) | 1 (0.3%) | 3 (0.8%) | 0 (0%) | 0 (0%) |
| *Enterococcus faecalis* | 44 (5.6%) | 27 (8.6%) | 10 (2.7%) | 3 (8.8%) | 4 (6.6%) |
| *Enterococcus faecium* | 26 (3.3%) | 15 (4.8%) | 10 (2.7%) | 0 (0%) | 1 (1.6%) |
| *Listeria monocytogenes* | 1 (0.1%) | 1 (0.3%) | 0 (0%) | 0 (0%) | 0 (0%) |
| *Candida albicans* | 10 (1.3%) | 2 (0.6%) | 6 (1.6%) | 0 (0%) | 2 (3.3%) |
| *Candida parapsilosis* | 2 (0.3%) | 0 (0%) | 1 (0.3%) | 0 (0%) | 1 (1.6%) |
| *Candida glabrata* | 1 (0.1%) | 0 (0%) | 0 (0%) | 1 (2.9%) | 0 (0%) |
| Other *Candida spp* | 3 (0.4%) | 0 (0%) | 1 (0.3%) | 1 (2.9%) | 1 (1.6%) |
