# Supplementary material for: Rapid bacterial identification and resistance detection using a low complexity molecular diagnostic platform in Zimbabwe
Source: PLOS Glob Public Health. 2025 Apr 9;5(4):e0004343. doi: 10.1371/journal.pgph.0004343 (PMC11981161; doi:10.1371/journal.pgph.0004343)
Supplement: S2 Table — *9 cultures also had Staphylococcus sp. (n=3), Klebsiella sp (n=5), Enterobacter sp (n=1) which were missed by BCID2. (DOCX) [file pgph.0004343.s006.docx]

S2 Table. Biofire off-panel organisms identified in blood culture positive, BCID2 negative samples

| Genus | Number of samples with organism |
| --- | --- |
| *Bacillus sp.* | 12 |
| *Corynebacterium sp.* | 11 |
| *Micrococcus sp* | 11 |
| *Acinetobacter sp.* | 8 |
| *Kocuria sp* | 8 |
| *Pseudomonas sp* | 3 |
| *Aerococcus sp* | 2 |
| *Yersinia sp* | 2 |
| *Brevibacillus sp* | 1 |
| *Brevibacterium sp* | 1 |
| *Dermabacter sp* | 1 |
| *Macrococcus sp* | 1 |
| *Microbacterium sp* | 1 |
| *Moraxella sp* | 1 |
| *Nocardia sp* | 1 |

**9 cultures also had Staphylococcus sp. (n=3), Klebsiella sp (n=5), Enterobacter sp (n=1) which were missed by BCID2*
