# Supplementary material for: Rapid bacterial identification and resistance detection using a low complexity molecular diagnostic platform in Zimbabwe
Source: PLOS Glob Public Health. 2025 Apr 9;5(4):e0004343. doi: 10.1371/journal.pgph.0004343 (PMC11981161; doi:10.1371/journal.pgph.0004343)
Supplement: S3 Table — *Excludes off-panel organisms; samples may contain none, one, or multiple organisms; ACBC: A. calcoaceticus-baumannii complex. (DOCX) [file pgph.0004343.s007.docx]

S3 Table. Status of false positive results: detection by BCID2 without identification of the organism using the reference methods

| **Organism** | **Number of false positive results** | **Organisms according to reference from same specimen*** |
| --- | --- | --- |
| *E. faecalis* | 16 | *E. faecium* (n=1), *E. coli* (n=2), *K pneumoniae* (n=6), *K. oxytoca* (n=1), *E. cloacae* (n=1), *Proteus* spp. (n=2), ACBC (n=1) |
| *E. faecium* | 15 | *E. faecalis* (n=5), *S. aureus* (n=1), *K. pneumoniae* (n=6), *K. oxytoca* (n=1), *E. cloacae* (n=2) |
| *S. aureus* | 10 | *E. faecalis* (n=2), *E. faecium* (n=2), coagulase-negative staphylococci (n=1), *E. coli* (n=2), *K. pneumoniae* (n=4), *Proteus* spp. (n=1) |
| *S. agalactiae* | 10 | *E. faecalis* (n=4), *E. coli* (n=1), *E. cloacae* (n=1) |
| *S. pneumoniae* | 2 | Coagulase-negative staphylococci (n=1), *K. pneumoniae* (n=1) |
| *A. calcoaceticus-baumannii* complex | 3 | *E. coli* (n=1), *K. oxytoca* (n=2), *K. pneumoniae* (n=1), *E. cloacae* (n=1), *S. marcescens* (n=1) |
| *E. cloacae* complex | 5 | *E. coli* (n=1), *K. oxytoca* (n=1), *K. pneumoniae* (n=4), *S. marcescens* (n=1) |
| *E. coli* | 5 | *K. pneumoniae* (n=3), *Proteus* spp. (n=2), coagulase-negative staphylococci (n=1) |
| *Salmonella* spp. | 1 | *E. faecium* (n=1) |
| *K. oxytoca* | 5 | *E. faecalis* (n=1), *K. pneumoniae* (n=5) |
| *K. pneumoniae* complex | 5 | *E. faecium* (n=1), *E. coli* (n=1) |
| *S. marcescens* | 1 | *E. faecalis* (n=1), *K. pneumoniae* (n=1) |
| *N. meningitidis* | 1 | - |
| *P. aeruginosa* | 1 | *E. faecalis* (n=1), *E. coli* (n=1), *K. pneumoniae* (n=1) |
| *S. maltophilia* | 1 | *E. faecalis* (n=1), *K. pneumoniae* (n=1) |

**excludes off-panel organisms; samples may contain none, one, or multiple organisms; ACBC: A. calcoaceticus-baumannii complex*
