# Supplementary material for: Rapid bacterial identification and resistance detection using a low complexity molecular diagnostic platform in Zimbabwe
Source: PLOS Glob Public Health. 2025 Apr 9;5(4):e0004343. doi: 10.1371/journal.pgph.0004343 (PMC11981161; doi:10.1371/journal.pgph.0004343)
Supplement: S4 Table — *Samples may contain none, one, or multiple organisms. (DOCX) [file pgph.0004343.s008.docx]

S4 Table. Status of false negative results: identification using the reference methods without detection by BCID2

| **Organism** | **Number of false negative results** | **Organisms detected by BCID2*** |
| --- | --- | --- |
| *E. faecalis* | 3 | *E. faecium* (n=1), *S. agalactiae* (n=1), coagulase-negative staphylococci (n=1); no organism detected (n=1) |
| *E. faecium* | 2 | *E. faecalis* (n=1), *Streptococcus* spp. (n=1), coagulase-negative staphylococci (n=1), *Salmonella* spp. (n=1) |
| *S. aureus* | 1 | *E. faecium* (n=1), coagulase-negative staphylococci (n=1), *Klebsiella pneumoniae* (n=1) |
| *S. agalactiae* | 1 | No organism detected |
| *A. calcoaceticus-baumannii* complex | 4 | *E. faecalis* (n=1), *S. agalactiae* (n=1), coagulase-negative staphylococci (n=2). |
| *E. cloacae* complex | 2 | Coagulase-negative staphylococci (n=1); no organism detected (n=1) |
| *E. coli* | 3 | *E. faecalis* (n=2), coagulase-negative staphylococci (n=1), *K. pneumoniae* (n=1) |
| *K. oxytoca* | 1 | No organism detected (n=1) |
| *K. pneumoniae* complex | 18 | *E. faecalis* (n=3), *S. agalactiae* (n=2), *S. pneumoniae* (n=1), coagulase-negative staphylococci (n=6), *S. aureus* (n=3), *E. coli* (n=1), no organism detected (n=12) |
| *Proteus spp.* | 2 | *E. faecalis* (n=1), coagulase-negative staphylococci (n=1), *S. aureus* (n=1), *E. coli* (n=1), no organisms detected (n=1) |
| *S. maltophilia* | 1 | No organisms detected (n=1) |

******* *samples may contain none, one, or multiple organisms*
