# Supplementary material for: Rapid bacterial identification and resistance detection using a low complexity molecular diagnostic platform in Zimbabwe
Source: PLOS Glob Public Health. 2025 Apr 9;5(4):e0004343. doi: 10.1371/journal.pgph.0004343 (PMC11981161; doi:10.1371/journal.pgph.0004343)
Supplement: Attachment — Submitted filename: AVAPAR Response to Reviewers_final.docx [file pgph.0004343.s012.docx]

**Response to Reviewers**

Manuscript revisions for the paper:

**Rapid bacterial identification and resistance detection in positive blood cultures from patients with sepsis in Harare, Zimbabwe: evaluation of a low complexity molecular diagnostic platform**

Dear Reviewers,

Thank you for the positive and constructive feedback you have provided. We appreciate the time you spent closely reading our manuscript and offering helpful suggestions for how and where to improve our paper. Your suggested revisions have strengthened the paper and improved clarity of our description of the research methodology, methods, and the contribution to knowledge through revision of the introduction and discussion sections.

Please see the table below for a description of the actions taken in response to each point raised.

| **Point raised** | **Author’s response** |
| --- | --- |
| **REVIEWER 1** |  |
| **Title** |  |
| Suggestion (Rapid bacterial identification and resistance detection using evaluation of a low complexity molecular diagnostic platform). | We have amended the title according to the suggestion and the new title is:  *Rapid bacterial identification and resistance detection using a low complexity molecular diagnostic platform in Zimbabwe* (Page 1) |
| **Abstract** |  |
| 1. Write in separate subtitle of abstract background, aim or objective of the study, methods, results, conclusions, and results.   Write meaningful and comprehensive key words of the research paper. Then author can add abbreviations. | We have amended the subtitles to the abstract according to the suggestion. (Lines 1 – 32)  We have revised the key words and added the abbreviation in the text:  ***Key words:*** *Blood stream infections; Sepsis; Antimicrobial resistance; Rapid diagnostics; Diagnostic accuracy; Multiplex PCR* (Page 1)  ***Abbreviations:*** *AMR – Antimicrobial resistance; LMIC – Low- and middle-income country; PCR – Polymerase Chain Reaction* (Page 1) |
| **Introduction** |  |
| 2. The information in introduction can discussed in details that include different detection methods in low and middle income countries compared with the low one. also the stages of development from past to date. | We have added a broad overview of methods used for pathogen detection and antimicrobial susceptibility testing discussing how methods developed and what are the differences between high- and low resource settings.  A paragraph was added reading:  ***“****Conventional microbiology traditionally relied on using phenotypic, manual methods for pathogen identification and antimicrobial susceptibility testing. With technological advances, high income countries have moved to using more rapid methods such as mass-spectrometry or molecular-based methods for pathogen identification and introduced automatisation in antimicrobial susceptibility testing. These changes resulted in reduced turnaround times and improved reliability and overall quality of results. Costs for these instruments and their consumables remain high and their operation requires advanced technical skills thus making them often unaffordable for low-resource settings.”* (Lines 67-74) |
| 3. If the author use these methods for detection of blood stream infections or sepsis, the author can write these methods in methodology paragraph in details. | The description of the Biofire platform was moved to the methods as suggested:  *“The multiplex PCR testing was conducted using the BCID2 which is a fully automated microbiological diagnostic assay that can detect 11 Gram-positive bacteria, 15 Gram-negative bacteria, 7 fungal pathogens, and 10 antimicrobial resistance genes.”* (Lines 116-118)  Additionally, we refer to the Biofire platform in a systematic review included in the introduction. A sentence was revised reading:  *“A recent systematic review including nine studies from the US, Europe, Australia and Asia showed high sensitivity and specificity of the BIOFIRE FILMARRAY Blood culture Identification 2 (BCID2, bioMérieux, Marcy l’Etoile, France) panel for species identification compared to culture-based methods.”* (Lines 80 – 83) |
| 4. These information can discussed in details that include different detection methods in low and middle income countries compared with the low one. also the stages of development from past to date (line 67-75). | We have added a broad overview of methods used for pathogen detection and antimicrobial susceptibility testing discussing how methods developed and what are the differences between high- and low resource settings.  A paragraph was added reading:  ***“****Conventional microbiology traditionally relied on using phenotypic, manual methods for pathogen identification and antimicrobial susceptibility testing. With technological advances, high income countries have moved to using more rapid methods such as mass-spectrometry or molecular-based methods for pathogen identification and introduced automatisation in antimicrobial susceptibility testing. These changes resulted in reduced turnaround times and improved reliability and overall quality of results. Costs for these instruments and their consumables remain high and their operation requires advanced technical skills thus making them often unaffordable for low-resource settings.”* (Lines 67-74) |
| 5. If the author use these methods for detection of blood stream infections or sepsis, the author can write these methods in methodology paragraph in details(line 67-75). | The description of the Biofire platform was moved to the methods as suggested:  *“The multiplex PCR testing was conducted using the BCID2 which is a fully automated microbiological diagnostic assay that can detect 11 Gram-positive bacteria, 15 Gram-negative bacteria, 7 fungal pathogens, and 10 antimicrobial resistance genes.”* (Lines 116-118)  Additionally, we refer to the Biofire platform in a systematic review included in the introduction. A sentence was revised reading:  *“A recent systematic review including nine studies from the US, Europe, Australia and Asia showed high sensitivity and specificity of the BIOFIRE FILMARRAY Blood culture Identification 2 (BCID2, bioMérieux, Marcy l’Etoile, France) panel for species identification compared to culture-based methods.”* (Lines 80 – 83) |
| **Methods** |  |
| 6. Write about type of the study and study participants. | We have revised the type of study and study participants in the text as follows:  *“This prospective single-centre validation study was conducted at Sally Mugabe Central Hospital (SMCH), Harare, Zimbabwe, a governmental referral centre with 1650 beds.”* (Lines 92-93)  *“All patients from the neonatal unit, paediatrics and adult wards with suspected blood stream infections who had positive blood cultures according to routine clinical and laboratory procedures between the 7th of June to the 10th of October 2022 were included in the study.”* (Lines 95 – 98) |
| 7. Show what are the inclusion and exclusion criteria of the study participants. | We have revised the text under study setting and participant inclusion to highlight the inclusion criteria of participants in this study and there were no exclusion criteria. The revised sentence is as follows:  *“All patients from the neonatal unit, paediatrics and adult wards with suspected blood stream infections who had positive blood cultures according to routine clinical and laboratory procedures between the 7th of June to the 10th of October 2022 were included in the study.”* (Lines 95 – 98) |
| 8. Enough to point to the specificity of the hospital only | We have revised the sentence in the text as follows:  *“This prospective single-centre validation study was conducted at Sally Mugabe Central Hospital (SMCH), Harare, Zimbabwe, a governmental referral centre with 1650 beds. The hospital serves more than half of the population of Harare and hosts the largest neonatal intensive care unit in the country.”* (Lines 92 - 95) |
| 9. Off point (line 89-91) | We have removed the statement about the laboratories acquisition of an automated blood culture machine.  We have decided to keep the sentence about the five trained laboratory scientist because it refers to the system usability survey component. Therefore, we have moved the sentence to the system usability section (Line 168) |
| 10. Write about blood culture diagnostic principals | We have added a few sentences on blood culture collection and transportation to the laboratory as follows:  *“Clinicians collected blood cultures in the wards according to the hospital’s standard operating procedures. Clinicians underwent regular blood culture collection technique training to reduce contamination and collect blood volumes required for the neonatal unit, paediatrics and adult blood culture bottles (1 – 2 ml, 3 ml and 8 – 10ml, respectively). Blood culture samples were delivered to the laboratory, which was on site, in less than 4 hours.”* (Lines 100 – 104) |
| 11. Mention the reactant and the product of the tests instead (line 103) | We have changed the subtitle title from BIOFIRE FILMARRAY BCID2 testing *to “Multiplex PCR testing”.* (Line 115) |
| 12. Then mention the reactant and the product of the tests instead (line 105-110) | We revised the description of the Biofire testing method, however, we could not include the exact reagents as they are proprietary and kept confidential by the manufacturers:  *“The multiplex PCR testing was conducted using the BCID2 which is a fully automated microbiological diagnostic assay that can detect 11 Gram-positive bacteria, 15 Gram-negative bacteria, 7 fungal pathogens, and 10 antimicrobial resistance genes.[7] Testing was performed according to the manufacturer’s instructions. Briefly, a hydration solution was loaded into the multiplex PCR panel pouch, and 0.2ml of the positive blood culture broth were mixed with the provided sample buffer. This mixture was added to the pouch and loaded onto the instrument which conducted nucleic acid extraction and multiplex nested PCR. A result was obtained within 60 min. For samples with invalid results or errors, repeat testing on the multiplex PCR panel was performed. Results were entered into a custom-made electronic database (Open-Data-Kit, ODK).”* (Lines 116 – 125) |
| 13. Rearrange data (line 125-148)   -These data for statistical analysis.  add the title below:  -preparation date for analysis.  then:  -data analysis.  -repeated data. add these data to study participant (system usability group)(line 150-151)  -better to write one principle for methodology which include reagent and system.(152-163) | We added subtitles to the data management and statistical analysis section as suggested (Lines 141 – 166)  This system usability section describes the methodology used for the usability survey of the Biofire. This involved a questionnaire that was administered to the laboratory scientists by a research assistant and did not involve the use of any reagents. We added a sentence to clarify this:  *“The survey questions were programmed onto ODK and were administered to the laboratory scientist by a research assistant.”* (Line 171 – 172) |
| 14. Those 5 participants who are staff member of the lab; author can add them as one of the study groups. | The study focuses primarily on the validation of the multiplex PCR in patients with blood stream infections and we wanted to assess the usability in addition to this validation. We separated the laboratory scientists from the study participants section because we feel that combining the laboratory scientists with the patients would make the methods section less clear to the reader. |
| **Discussion** |  |
| 15. Please discuss the results of current study in more details   Put the results in categorized paragraphs include result of current study, discussion of result then comparison with other globally researches  Avoid repeating data and information.  Avoid writing in self-expression., and do not confirm the result by your self-according to the high quality of techniques of staff, but confirming of results is by the matching and comparing the current results with previous studies. | The discussion section was revised according to the suggestion. (Lines 288 – 387)  Paragraphs were reorganised to fit better thematically. They now discuss the following broad categorised themes - pathogen and resistance detection and sepsis, test performance, contamination, test usability, considerations on implementation in low-resource settings. The sections discuss the study findings followed by contextualisation in the broader literature. We have also removed repetitions. The discussion section was also revised as suggested and rephrased to avoid self-expression. |
| 16. Better to write the active ingredient of all antibiotics(line 270) | “Third generation cephalosporin resistance” is a broad designation referring to resistance to ceftriaxone, cefotaxime and/or ceftazidime which is widely used in reporting AMR surveillance data. This term is also used by the WHO when defining priority pathogens of critical importance to human health  <https://iris.who.int/bitstream/handle/10665/376776/9789240093461-eng.pdf?sequence=1> |
| 17. Delete the repeated data (line 355-358) | We have deleted the repeated data at suggested. |
| 18. Put discussion, conclusion, recommendations and limitations all in separated paragraph. | We have added the subtitles in the discussion section as suggested. (Lines 288 – 387) |
| 19. Summarize the acknowledgment | We summarized the acknowledgements section as follows:  *We would like to extend our gratitude to the research team for implementing the study protocol. We would like to thank the Sally Mugabe Central Hospital Microbiology laboratory scientists for conducting blood culture testing. Additionally, we thank the laboratory scientists from the National Reference Laboratory (Zimbabwe), IHMA Europe (Switzerland), Ares Genetics GmbH (Austria) and Microbes Ng (United Kingdom) for conducting further tests on the study isolates. Importantly, we would like to thank the clinicians who supported and participated in the blood culture collection training.* (Lines 390 – 396) |
| **REVIEWER 2** |  |
| 1. The title is long therefore requires rephrasing | We have revised the title and the new title is:  *Rapid bacterial identification and resistance detection using a low complexity molecular diagnostic platform in Zimbabwe* (Page 1) |
| 2. Under the subsection study setting and participant inclusion | We have revised the text under study setting and participant inclusion to highlight the inclusion criteria of participants in this study and there were no exclusion criteria. The revised sentence is as follows:  *“All patients from the neonatal unit, paediatrics and adult wards with suspected blood stream infections who had positive blood cultures according to routine clinical and laboratory procedures between the 7th of June to the 10th of October 2022 were included in the study.”* (Lines 95 – 98) |
| 3. It is not clear if participants were enrolled and blood samples taken from them or if samples were stored. The authors need to clearly write that section. | We did not enrol participants, however, we tested routinely collected samples received from all patients from the neonatal unit, paediatrics and adults’ wards in real time.  The revised sentence is as follows:  *“All patients from the neonatal unit, paediatrics and adult wards with suspected blood stream infections who had positive blood cultures according to routine clinical and laboratory procedures between the 7th of June to the 10th of October 2022 were included in the study.”* (Lines 95 – 98)  Reference testing in the National Reference Laboratory and the overseas laboratories was done on stored samples:  *All positive sub-cultures were inoculated in tryptone soya broth prepared with 20% glycerol (TSB) and stored at -20 degrees for reference testing. (Lines 111 – 112)* |
| 4. Do the readers understand reference testing/ please define what is referred to as reference testing | We added a definition for a reference test as follows:  *A reference test, also referred to as a gold standard test, is used to discriminate patients with blood stream infections and those without evidence of blood stream infections. (*Line 112 – 114) |
| 5. Under the subsection of data management the statement “Sample size was determined by the available number of BCID2 panel cartridges” seems out of place therefore should be removed . | We have removed the sample size sentence as suggested |
